# Developer and Partnership Differences in COVID-19 and Other Infections: Insights from DNA Vaccines

**DOI:** 10.3390/jmahp12040025

**Published:** 2024-10-29

**Authors:** Ryo Okuyama

**Affiliations:** College of International Management, Ritsumeikan Asia Pacific University, Beppu 874-8577, Japan; ryooku@apu.ac.jp

**Keywords:** DNA vaccine, clinical development, developer, partnership, biopharmaceutical company, public institution

## Abstract

Historically, vaccine development has been heavily supported by government and public institutions. On the other hand, private biopharmaceutical companies have played a significant role in the development of innovative new therapies using novel pharmaceutical technologies. COVID-19 vaccines using new vaccine technologies, such as mRNA and adenoviral vectors, were rapidly developed by emerging biopharmaceutical companies in collaboration with large corporations and public organizations. This underscores the crucial role of emerging biopharma and public–private partnerships in advancing new vaccine technologies. While these innovations have been suggested as models for future vaccines, their applicability to other infectious diseases requires careful assessment. This study investigated the characteristics of the developers and partnerships in the development of DNA vaccines as a next-generation vaccine platform. The analysis revealed that while emerging biopharmaceutical companies and private–private and private–public partnerships were crucial during the COVID-19 pandemic, public organizations and public–public collaborations primarily led to the clinical development of vaccines for other diseases. Strategies for vaccine development using new vaccine technologies should be tailored to the specific characteristics of each disease.

## 1. Introduction

Owing to its significant impact on public health, vaccine research and development (R&D) has received substantial support from governments and public organizations in many countries. In the United States, government agencies have historically made large investments in vaccine R&D [1]. The significant contribution of the public sector to vaccine development has been reported for vaccines against human immunodeficiency virus (HIV) [2], human papillomavirus (HPV) [3], and Ebola virus [4]. Incumbent pharmaceutical companies have also played an important role in vaccine development. While various public and private biotechnology organizations have participated in the early development of vaccines, about 40% of the vaccines under phase II clinical trials were produced by the “big four” pharma (GlaxoSmithKline (London, UK), Merck & Co. (Rahway, NJ, USA), Pfizer (New York City, NY, USA), and Sanofi Pasteur (Lyon, France)) as of 2018, prior to the coronavirus disease 2019 (COVID-19) pandemic [5].

The development of COVID-19 vaccines has reshaped the above landscape. The first vaccines to receive emergency use authorization and be administered worldwide were two mRNA vaccines (Spikevax, Comirnaty) and one adenoviral vector vaccine (Vaxzevria) [6]. Notably, mRNA vaccines were the first to receive approval as pharmaceuticals for COVID-19 [7], while adenoviral vector vaccines had previously only received approval against the Ebola virus around the same time [8]. The practical implementation of these new vaccine technology platforms marked an epoch-making event in COVID-19 vaccine development, showcasing a compelling example of the importance of advancing pharmaceutical technologies. Many of these cutting-edge pharmaceutical technologies are based on research outcomes from public research institutions, such as universities, and are pursued for practical application by young biopharmaceutical companies, including university startups [9,10]. Spikevax and Comirnaty were developed by the biopharmaceutical companies Moderna (Cambridge, MA, USA) and BioNTech (Mainz, Germany), respectively, both founded around 2010 [6]. Vaxzevria was developed by Vaccitech (Harwell, UK), a bio-startup spun out from the University of Oxford, with support from AstraZeneca (Cambridge, UK) [6]. Thus, the rise of small biopharmaceutical companies, recently known as “emerging biopharma” [11], in the development of new vaccine platforms was a significant trend in COVID-19 vaccine development [12,13].

Another significant feature of COVID-19 vaccine development was the collaboration between public and private sectors for the efficient advancement of vaccine development. Unprecedented financial support from the government, novel technology development by startups, and effective partnerships between private companies and between public and private sectors have synergistically accelerated vaccine development [14,15,16,17]. In response to these features observed in COVID-19 vaccine development, many researchers are emphasizing the importance of investing in new vaccine platform technologies and companies and the significance of private–private and public–private partnerships in future vaccine development [18,19,20,21,22].

However, COVID-19 has been an unprecedented pandemic characterized by rapid global spread with high mortality rates over a short period, severely disrupting human and material movement and contact, with significant economic and societal impacts [23]. This situation raised questions about whether the unique features of COVID-19 vaccine development can be directly applied to the future development of vaccines against other infectious diseases using new vaccine platform technologies. Moreover, COVID-19 vaccines have achieved significant commercial success [24], highlighting a distinct situation compared with other infectious disease vaccines.

The objective of this study is to analyze and examine whether the characteristics of vaccine developers and partnerships observed during COVID-19 will become a trend for future vaccine development or if they are unique observations specific to COVID-19 that may not apply to other infectious disease vaccines. By doing so, this study aims to provide a platform for stakeholders, such as companies and governments, to discuss future vaccine development strategies. The results of this study may offer insights for pharmaceutical companies to consider technology and partnership strategies for future infectious disease vaccine development. Additionally, it may provide resources for policymakers to consider measures to support infectious disease vaccine development.

To explore this issue, this study examined differences in developer and partnership dynamics between COVID-19 and other infectious disease vaccines using DNA vaccine technology. DNA vaccines were being studied in clinical trials well before the first clinical studies with mRNA vaccines were conducted. No DNA vaccines have been approved globally yet, and many DNA vaccine candidates against various pathogens have undergone clinical testing. Therefore, this technology is pivotal in forecasting future vaccine development trends using new technology platforms. Using clinical trial database information, this study analyzed the profiles of clinical trial sponsors and collaborators involved in DNA vaccine development. The analysis highlights that the rise of young biopharmaceutical companies (less than 30 years old) and the activation of private–private and private–public partnerships observed during COVID-19 vaccine development were phenomena specific to COVID-19 and had different characteristics and trends from those observed for other infectious disease vaccines.

## 2. DNA Vaccine

A DNA vaccine is a vaccine technology platform that uses part of the pathogen’s DNA to generate immunity [25]. DNA vaccines have advantages over other vaccine platforms, including scalability, stability, ease of manipulation, and suitability for stockpiling [26]. To date, DNA vaccines against several pathogens, such as HIV, Ebola, HPV, Zika, and SARS-CoV-2, have been investigated [27]. However, although the DNA vaccine ZyCoV-D targeting COVID-19 has been authorized for emergency use in India, no DNA vaccines have yet been approved globally [28]. DNA vaccines, like mRNA and adenoviral vector vaccines, could play a role in future vaccines [28].

## 3. Data Collection and Analysis

### 3.1. Clinical Trials of DNA Vaccines for Infectious Diseases

All ongoing clinical trials of DNA vaccines as of 24 April 2024, were compiled by searching “DNA vaccine” in the “other term” field on ClinicalTrials.gov (https://clinicaltrials.gov/ (accessed on 24 April 2024)). This comprehensive website includes studies conducted across all 50 states and over 200 countries and has been widely used for analyzing trends in clinical development. By reviewing each DNA vaccine’s name from the descriptions under the “Interventions” section and indication under the “Conditions” section on the site, the analysis revealed that a total of 119 DNA vaccines targeting infectious diseases were under development. Among these, 18 vaccines targeted COVID-19, 46 targeted HIV, and 15 targeted influenza. Other indications included fewer than ten vaccines in development: nine for Hepatitis B, four each for HPV and Herpes simplex virus (HSV), and three or fewer for other indications. The distribution of clinical development stages identified from the above website information and infectious disease indications of the 119 vaccines are summarized in Table 1.

### 3.2. Developer Types of DNA Vaccines in Different Indications

For these 119 vaccines, the organizations listed under the “Sponsor” section (private companies, federal organizations, universities, public research institutions, hospitals, and public networks) were identified as developers of each DNA vaccine. Notably, in some cases, multiple developers were listed for each vaccine, resulting in the total number of developers exceeding 119. These developers were then classified into emerging biopharmaceutical (EBP), large pharmaceutical (LP), and public organization (federal institutions, universities, public research institutions, hospitals, and public networks). An EBP is defined as a biopharmaceutical company with yearly sales of less than USD 500 million [11]. Companies with revenues exceeding USD 500 million in 2023 were identified from a website that reported the revenues of the top 100 pharmaceutical companies in 2023 (https://www.chemanalyst.com/ChemAnalyst/PharmaCompanies (accessed on 24 June 2024)). Companies not included in this category were classified as EBP, whereas those included in this category were classified as LP. Given that the EBP status is defined by revenue, it includes small companies that were relatively long-established and did not meet the revenue criteria. Emerging medical technologies often originate from academic research at universities, leading to the establishment of biopharmaceutical companies that advance nascent technologies toward product readiness. Therefore, it is crucial to distinguish between these young biopharmaceutical companies that drive this process and established companies with relatively low sales. However, an EBP classification based solely on revenue may include both, thereby limiting accurate analysis. To avoid this limitation, the founding year of each EBP was examined, and the EBPs were classified into companies established less than 30 years ago (EBP < 30 y) and those established 30 years or earlier (EBP ≥ 30 y). The rationale for selecting 30 years as the cut-off is that pharmaceutical technologies show an S-curve life cycle from their nascent to commercialization phase [29], spanning approximately 30 years for monoclonal antibody medicine [30] and gene therapy [31]. The names of EBPs, their founding years, employee counts, and the number of vaccines developed by each EBP in this dataset are provided in the Appendix A. The analysis examined how the distribution of classified developer types varies across different indications, as presented in Table 2. Indications with fewer than ten DNA vaccines in development were grouped under “Others”.

EBPs < 30 y were the major developers only for COVID-19 (10/19 developers). For other infectious disease vaccines, public organizations dominated as developers (45/51 developers in HIV, 8/15 developers in influenza, and 24/41 developers in “others” categories). The distribution of developer types for COVID-19 significantly differed from that for HIV, influenza, and “others” categories (*p* < 0.01 in HIV, *p* < 0.05 in influenza, and *p* < 0.05 in “others” in the chi-square test). These data indicate that the rise of young biopharmaceutical companies in vaccine development using new platform technologies such as DNA vaccines is a phenomenon specific to COVID-19. For other infectious diseases, traditional public organizations remain the primary drivers of DNA vaccine development.

### 3.3. Partnership Pattern of DNA Vaccines in Different Indications

Partnerships between private companies and the public and private sectors have played a significant role in the rapid development of COVID-19 vaccines using new vaccine platforms. Therefore, the partnership patterns in DNA vaccine development were further examined for each indication. In the clinical trial information on ClinicalTrials.gov (accessed on 24 April 2024), alongside sponsors, collaborators are described under the section “Collaborator”. DNA vaccines with no collaborators were categorized as having “no partnership”. For DNA vaccines where organization names were listed under “Collaborator”, the attributes of all organizations listed under Sponsor and Collaborators were examined. If only private companies were listed as Sponsor and Collaborators, the collaboration was categorized as a “private–private” partnership; if both private companies and public organizations were listed, it was categorized as a “private–public” partnership. If only public organizations were listed, the collaboration was categorized as a “public–public” partnership. The distribution of partnership patterns for each indication is presented in Table 3.

For COVID-19, private–public partnership was dominant among all collaborations (7/11 collaborations), with no public–public partnerships. In contrast, public–public partnership was dominant for HIV (22/32 collaborations). In the case of influenza, few vaccines utilized partnerships (2/15 vaccines). For more than half of “other” infectious diseases, partnerships were not established (23/40 vaccines), with an almost equal distribution of private–public and public–public partnerships (7 and 6, respectively). The distribution of partnership patterns significantly differed between COVID-19 and HIV as well as between COVID-19 and influenza (*p* < 0.01 and *p* < 0.05 using the chi-square test, respectively). The distribution pattern for “others” was not significantly different from that observed in COVID-19, but the *p* value was close to 0.05, indicating that the distribution of partnership patterns between the two categories tends to be different (*p* = 0.065 in the chi-square test).

## 4. Discussion

This analysis demonstrates that developer types and partnership patterns for DNA vaccines, a next-generation vaccine platform, differ between COVID-19 and other infectious diseases. For COVID-19 vaccine development, EBP represented the core of developers, with a notable influx of young biopharmaceutical companies established within the last 30 years. In contrast, public organizations dominated as developers of vaccines against other infectious diseases. Prior to the COVID-19 pandemic, vaccine development was widely recognized for its heavy reliance on the involvement of public organizations, a trend that has continued in infectious diseases other than COVID-19 in recent years. The higher participation of EBP, specifically in COVID-19 vaccine development, is likely attributed to substantial economic expectations. Specifically, sales of COVID-19 vaccines totaled USD 49 billion in 2021 for Spikevax, Comirnaty, and Vaxzevria combined, and Comirnaty was the highest-selling pharma product globally in the year [24]. These vaccines utilize new vaccine platforms, including mRNA and adenoviral vectors, indicating that the substantial market potential of COVID-19 vaccines likely accelerated the entry of young EBPs in the field of new vaccine platforms.

In addition, all of these new vaccine platform technologies, such as mRNA, adenoviral vectors, and DNA therapeutics, can be applied to various disease indications other than infectious vaccines. mRNA therapeutics have been developed for cancer, rare diseases, and cardiovascular diseases [32,33]. Adenoviral vectors and DNA therapeutics have been tested in various gene therapies other than vaccination [34,35]. Considering the market potential and drug pricing, EBPs developing drugs using these new technologies may be motivated to prioritize their applications for diseases other than infectious diseases besides COVID-19. Moreover, private companies are highly involved in COVID-19 vaccine development, leading to frequent private–private and private–public partnerships. This trend was similar to that observed during DNA vaccine development. In contrast, for infectious diseases other than COVID-19, private–private or private–public partnerships were not predominant in DNA vaccine development. Instead, collaboration between public organizations or development without partnerships was more common. Thus, the entry of EBPs in the field of new vaccine platforms and the activation of private–private and public–private partnerships are relatively specific phenomena observed in COVID-19 vaccine development. Whether these trends will manifest similarly in the future development of vaccines against other infectious diseases is challenging to assert. Owing to the success of mRNA and adenoviral vector vaccines, the phenomena observed in COVID-19 vaccine development have been frequently proposed as guidance for future vaccinology. However, whether the trends observed in COVID-19 vaccine development can be directly applied to other infectious disease vaccines, given the unprecedented impact of COVID-19 on the markets and society, must be carefully considered.

The trends observed in the development of DNA vaccines are likely to be applicable to other vaccine technology platforms in the future. Currently, various new vaccine-related technologies, including universal vaccines and next-generation mRNA medical technologies, are being developed. Although vaccine development by emerging biopharmaceuticals actively pursuing advanced technologies is influenced by commercial incentives, public support will continue to be essential, especially for combating infections with low incidence rates and in low-income countries. Conversely, in the case of a global pandemic akin to COVID-19, the contribution of advanced technology development by emerging biopharmaceuticals can be strongly anticipated.

This study is limited by its small sample size. Consequently, infections other than COVID-19, HIV, and influenza were grouped together and analyzed as “others”. Although it is not possible to increase the sample size any further due to the comprehensive nature of the survey, it should be noted that a larger dataset is necessary for more detailed analysis. Another limitation of this study is that DNA vaccines have not yet convincingly proven their efficacy in humans. If the clinical efficacy of DNA vaccines is demonstrated in the future, large pharmaceutical companies may show increased interest in investing, which could lead to changes in developer types and partnership patterns. It is important to note that the insights from this study are based on the current state of DNA vaccine development.

This study highlights the importance of tailoring the strategies of vaccine development using new pharmaceutical technologies to match the specific requirements of each target disease, considering the medical-economic feasibility and societal impact of each infectious disease. This perspective provides new insight into vaccine development and market access, and the health policies that influence them.

## Figures and Tables

**Table 1:** Distribution of clinical development stages and infectious disease indications of 119 DNA vaccines.

	Clinical Development Stage							
Indication	Phase 1	Phase 1, 2	Phase 1, 2, 3	Phase 2	Phase 2, 3	Phase 3	Phase Undis-Closed	Total
Allergic rhinitis	1			1				2
Andes virus	1							1
COVID-19	7	7	3	1				18
Cytomegalovirus	2							2
Dengue virus	2							2
Ebola virus	1							1
Hantaan virus	1	2						3
Hepatitis B virus	5	1		2			1	9
Hepatitis C virus	2	1						3
HIV	30	11		4			1	46
HPV	3	1						4
HSV	2	1		1				4
Influenza	15							15
Malaria	2	1						3
MARS		1						1
Pulmonary tuberculosis	1							1
VEE virus	2							2
Zika virus	1	1						2

HIV: human immunodeficiency virus, HPV: human papillomavirus, HSV: herpes simplex virus, MARS: Middle East respiratory syndrome coronavirus, VEE: Venezuela equine encephalitis.

**Table 2:** Developer-type distribution in each infectious indication in DNA vaccines under development.

	Type of Developer				Significance
Indication	EBP < 30 y	EBP ≥ 30 y	LP	Public	*p*-Value (vs. COVID-19)
COVID-19	10	4	0	4	NA
HIV	2	3	1	45	<0.01
Influenza	2	5	0	8	<0.05
Others	9	6	2	24	<0.05

“Others” includes allergic rhinitis, Andes virus, cytomegalovirus, dengue virus, Ebola virus, Hantaan virus, hepatitis B virus, hepatitis C virus, human papillomavirus virus, herpes simplex virus, malaria, Middle East respiratory syndrome coronavirus, pulmonary tuberculosis, Venezuela equine encephalitis virus, and Zika virus. The statistical difference in the developer-type distribution between COVID-19 and HIV, COVID-19 and influenza, and COVID-19 and “others” was tested using the chi-square test, respectively. COVID-19: coronavirus disease 2019, EBP: emerging biopharmaceutical, LP: large biopharmaceutical, HIV: human immunodeficiency virus, NA: not applicable.

**Table 3:** Distribution of partnership pattern in each infectious indication in DNA vaccines under development.

	Pattern of Partnership				Significance
Indication	Private–Private	Private–Public	Public–Public	No Partnership	*p*-Value (vs. COVID-19)
COVID-19	4	7	0	7	NA
HIV	1	9	22	14	<0.01
Influenza	1	1	0	13	<0.05
Others	4	7	6	23	0.065

“Others” includes allergic rhinitis, Andes virus, cytomegalovirus, dengue virus, Ebola virus, Hantaan virus, hepatitis B virus, hepatitis C virus, human papillomavirus virus, herpes simplex virus, malaria, Middle East respiratory syndrome coronavirus, pulmonary tuberculosis, Venezuela equine encephalitis virus, and Zika virus. The statistical difference in the partnership pattern distribution between COVID-19 and HIV, COVID-19 and influenza, and COVID-19 and “others” was tested using the chi-square test, respectively. COVID-19: coronavirus disease 2019, HIV: human immunodeficiency virus, NA: not applicable.

## Data Availability

The original contributions presented in the study are included in this article; further inquiries can be directed to the corresponding author.

## Appendix A. The Names of EBPs, Their Founding Years, Employee Counts, and the Number of Vaccines Developed by Each EBP in This Dataset

**Company Name**	**Founding Year**	**Employee Counts**	**# of Vaccines Developed in This Dataset**
**EBP < 30 y**			
Alvea	2019	59	1
AnGes	1999	62	2
Entos Pharmaceuticals	2016	70	1
Genexine	1999	127	3
Immuno Cure	2020	11–50	1
Immunomic Therapeutics	2005	30	1
Newish Technology	2014	<25	1
Nykode Therapeutics	2006	179	1
PharmaJet	2005	33	1
PowderMed	2004	39	5
Scancell	1997	51	1
Shenzhen Immuno Cure Biomedical	2015	100	1
SL VAXiGEN	2017	14	1
Takis	2013	37	1
Tripep	1997	<50	1
Worcester HIV Vaccine	2018	<50	1
**EBP ≥ 30 y**			
Epimmune	1993	150	1
GeneOne Life Science	1976	60	4
Ichor Medical Systems	1994	<25	1
Imunon	1982	33	1
Inovio Pharmaceuticals	1979	122	8
Vical	1987	<50	3
The founding years and employee counts were identified from each company’s website, as well as from Pitchbook (https://pitchbook.com/), Crunchbase (https://www.crunchbase.com/), Zoominfo (https://www.zoominfo.com/), Synapse (https://synapse.patsnap.com/), Bioworld (https://www.bioworld.com/), and Stockanalysis (https://stockanalysis.com/).			
